# Fabrication of Nanoshell-Based 3D Periodic Structures by Templating Process using Solution-derived ZnO

**DOI:** 10.1186/s11671-017-2186-6

**Published:** 2017-06-17

**Authors:** Shinji Araki, Yasuaki Ishikawa, Xudongfang Wang, Mutsunori Uenuma, Donghwi Cho, Seokwoo Jeon, Yukiharu Uraoka

**Affiliations:** 1 Graduate School of Materials Science, Nara Institute of Science and Technology, 8916-5 Takayama, Ikoma, Nara 630-0192 Japan; 2 Department of Materials Science and Engineering, Korea Advanced Institute of Science and Technology, Daejeon, 305-701 Republic of Korea

**Keywords:** Solution-derived ZnO, Three-dimensional (3D), Nanoshell-based structure, Hierarchical architecture, Proximity field nanopatterning, Templating process

## Abstract

Fabrication methods for a 3D periodic nanostructure with excellent and unique properties for various applications, such as photonic and phononic crystals, have attracted considerable interest. Templating processes using colloidal crystals have been proposed to create nanoshell-based 3D structures over a large area with ease. However, there are technical limitations in structural design, resulting in difficulties for structural flexibility. Here, we demonstrate a combination of proximity field nanopatterning and infiltration processes using solution-derived ZnO for a nanoshell-based 3D periodic structure with high structural flexibility and controllability. A unique process of infiltration of a solution-derived material into a polymeric template prepared by a proximity field nanopatterning process achieves the fabrication of a pre-formed layer that works as a protective layer for the template and framework for the inverse structure. Subsequently, this process shows the controllability of nanoshell thickness and significant improvement in the structure height shrinkage factor (16%) compared to those of a previous non-vacuum infiltration method (34%). The proposed method offers high controllability and flexibility in the design of structural sizes, leading to further development toward nanoshell-based 3D structures for various applications including energy devices and sensors.

**Electronic supplementary material:**

The online version of this article (doi:10.1186/s11671-017-2186-6) contains supplementary material, which is available to authorized users.

## Background

Three-dimensional (3D) periodic nanostructures have received much attention due to their excellent and unique properties. The potential for this technology is a rapidly developing field that shows promise in various applications including photonic crystals (PhCs) [1–3], phononic crystals (PnCs) [4], battery materials [5, 6], and microfluid channels [7]. Research has shown that structural periodicity can be achieved by using various fabrication methods [8–11] with high controllability and flexibility, which are very important characteristics for this technology. The templating process provides a simple procedure compared with traditional bottom-up approaches, using a 3D periodic structure as a template composed of self-assembled colloidal spheres or a photopolymer followed by infiltration of inorganic materials and template removal [9, 12–19]. Although vacuum processes with atomic layer deposition (ALD) followed by chemical vapor deposition (CVD) have been used as infiltration techniques, an excess overlayer—which forms on the template after infiltration—will require additional reactive ion etching (RIE) to remove it [9, 18, 19]. In contrast, a non-vacuum process with electrodeposition [7, 12, 20] and sol-gel reaction [13, 21–23] provides the creation of fine inverse structures and has demonstrated the optical properties of resultant 3D periodic structures composed of ZnO, Cu_2_O, and TiO_2_ [13, 20, 23]. Furthermore, non-vacuum processes have the advantages of cost-effectiveness and shorter processing time.

ZnO is a promising semiconductor material with outstanding optical and electrical properties. Moreover, chemical and thermal stability [24] make ZnO an excellent candidate for various applications such as PhCs, sensors, and transparent electrodes [13, 14, 24]. Additionally, Al-doped ZnO has also shown high performance in the field of thermoelectric devices without the need for toxic or rare elements [25, 26].

Meanwhile, artificially nanostructured materials have attracted considerable attention as a means of fabricating nanostructures with unique properties. For example, Biswas et al. suggested that a 3D hierarchical architecture with micro- and nanostructures contributes strongly to a reduction in thermal conductivity leading to a significant improvement in thermoelectric performance [27]. This indicates that a fabrication method with remarkable versatility and simplicity is highly desirable for the preparation of well-ordered 3D hierarchical architectures, in order to enhance and manage various material characteristics. Among the architectures, a hierarchical one consisting of nanoshell structures fabricated by the templating process [28–31] has been the focus of attention due to the extremely high surface-to-volume ratio unlike traditional inverse structures. However, the ALD technique requires vacuum pumps with high energy consumption and expensive equipment; both of which are necessary for the infiltration process to create a nanoshell-based 3D periodic structure [28, 29]. Consequently, infiltration using non-vacuum processes has the technical difficulty in obtaining controllability of nanoshell thickness, because it densely infiltrates a solution-derived material into a 3D template to create monolithic frameworks for a freestanding inverse structure [20, 23, 31]. To date, this emerging field lacks substantial reports on fabrication methods for nanoshell-based 3D periodic structures using a consistent non-vacuum process.

In this paper, we demonstrate a combination of a proximity field nanopatterning (PnP) process [32–36] and infiltration process with solution-derived ZnO, in order to create a nanoshell-based 3D periodic structure. PnP is a method to form 3D polymeric periodic structures utilizing a 3D intensity distribution of light into a photopolymer, generated by the passage of light through a phase shift mask with periodic relief patterns. This process has high flexibility in the design of structural sizes by changing the relief pattern because the intensity distribution depends strongly on its design. Thus, this process can resolve the problems of templating process from colloidal self-assembly such as difficulty in structural modification and non-uniformity caused from defects [30]. The purpose of this study is to fabricate a nanoshell-based 3D hierarchical architecture by a consistent non-vacuum method using solution-derived ZnO. Moreover, this study evaluated the shrinkage factors of 3D ZnO periodic structures fabricated by the polymeric templating process.

## Methods

### Preparation of 3D Polymeric Template by PnP

A cover glass (thickness 0.16–0.19 mm) cleaned with oxygen plasma for 2 min was used as a substrate. A bilayer film was prepared on the substrate to prevent nanostructured membrane delamination during the development process. Detailed information on the procedures for 3D polymeric templates is described in literatures [28, 32, 33, 37]. Firstly, a negative-tone photoresist (SU-8, MicroChem) flood-exposed to UV light was formed on the substrate as an adhesion layer (<2 μm). The photoresist film with a 10 μm thickness was then spin-coated (2000 rpm for 30 s) on the adhesion layer. The substrate-prepared bilayer film was subsequently soft-baked (95 °C for 10 min) on a hot plate. The phase shift mask used in this study was made from poly(dimethylsiloxane) (PDMS) (VDT-731, HMS-301, Gelest), which has a square-arrayed relief pattern composed of cylinders (periodicity 600 nm, diameter 480 nm, relief depth 420 nm). The PDMS phase shift mask was directly contacted with a top surface of the photoresist film during UV irradiation. After UV irradiation (wavelength 355 nm) through the phase shift mask using an Nd:YAG laser (Awave355-300mW40K, Advanced Optowave) with a beam expander and a collimator, the sample was post-exposure-baked (65 °C for 7 min) on a hot plate. The unexposed regions were then removed using propylene glycol methyl ether acetate (PGMEA) (SU-8 developer, MicroChem) followed by rinsing with ethanol so that a 3D polymeric template was obtained [32–36].

### Fabrication of Nanoshell-Based 3D Structure

**Fig. 1:**
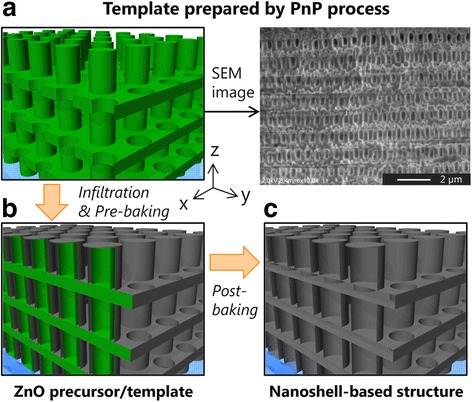
Schematic diagram of the procedure using infiltration process with solution-derived ZnO for nanoshell-based 3D structures. **a** Preparation of template by PnP process. **b** Infiltration of ZnO precursor solution into the template and pre-baking several times, and **c** Post-baking for template removal

Figure 1 shows a schematic diagram of the procedure using the infiltration process with solution-derived ZnO for nanoshell-based 3D periodic structures. A ZnO precursor solution (2.0 M) composed of a metal organic decomposition (MOD) material (SYM-Zn20, Kojundo Chemical Lab.) was used as an infiltrating material. Initially, a few drops of the precursor solution were deposited so that the entire top surface of the 3D polymeric template prepared by PnP was covered. The sample was then spin-coated at 2000 rpm for 20 s to achieve uniformity of the solution supply. Next, vacuum degassing was carried out to assist the penetration into the bottom of the template, as used commonly in the fabrication of dye-sensitized solar cells (DSSC) [38, 39]. The pre-baking was finally done in an electric furnace (FO310, Yamato Scientific) at 220 °C for 1 h in oxygen atmosphere (flow rate 14 L/min). The temperature condition used on the gel state of the ZnO precursor was determined in reference to our previous study [40]. Furthermore, the procedure from spin-coating to pre-baking was performed several times in order to examine the cycle number dependence of the infiltration process on the resultant structural properties.

To obtain a 3D ZnO structure by using the templating process, template removal is required after the infiltration process. On top of this, due to insufficient heat treatment, removal of the remaining ZnO precursor that infiltrated into the template is also necessary. Thus, post-baking serves a dual purpose: primarily, to remove the template and, consequently, to contribute to the pyrolysis of the precursor to serve as preparation for the ZnO. In this light, post-baking was done on the precursor-infiltrated template at 410 °C for 4 h in oxygen atmosphere [19]. Our previous research has shown that the pyrolysis temperature of the precursor is above 360 °C [40]. As mentioned above, this procedure has two different baking processes because the post-baking at temperatures greater than 400 °C after infiltration without pre-baking causes a structural collapse (Additional file 1: Figure S1a, b).

### Characterization

The morphologies of 3D periodic structures fabricated by PnP and infiltration of solution-derived ZnO were observed by scanning electron microscopy (SEM) (SU-6600, Hitachi). We evaluated the structural periodicity, quality, and uniformity in obtained structures for each fabrication step by cross-sectional SEM images. Additionally, the shrinkage factors are estimated by measurements of the structural size differences between resultant ZnO structures and the templates prepared by PnP. Moreover, energy-dispersive X-ray spectrometry (EDX) analysis was conducted to confirm the removal of the template and to identify a composition ratio of solution-derived ZnO, using the same apparatus with an accelerating voltage of 5.0 kV. To measure reflectance spectra of the polymeric template and the nanoshell-based 3D periodic structure, UV-Vis spectroscopy (V-570, JASCO) was used. Furthermore, the bandgap energy of the solution-derived ZnO was estimated experimentally by measuring the transmission spectrum.

## Results and Discussion

Structural sizes in 3D ZnO structures obtained through templating processes depend highly on the sizes of the primary template. The repeated periodicity in out-of-plane direction (*z* axis) in structures, called the Talbot distance (*Z*_T_), prepared by PnP can be calculated by using the following formula [41].

$$ {Z}_{\mathrm{T}}=\frac{\raisebox{1ex}{${\lambda}_0$}\!\left/ \!\raisebox{-1ex}{${n}_{\mathrm{m}}$}\right.}{1-\sqrt{1-{\left(\frac{\lambda_0}{n_{\mathrm{m}}\cdot p}\right)}^2}} $$

The formula is composed of an irradiation light with a wavelength in free space *λ*_0_, the refractive index of the medium *n*_m_, and relief pattern periodicity *p*. In this study, the ideal Talbot distance was calculated using the formula with the parameters: *λ*_0_ = 355 nm, *n*_m_ = 1.66, and *p* = 600 nm. Table 1 shows the Talbot distances of the theoretical value and a measured value from the prepared SU-8 template.

**Table 1:** Calculated and measured Talbot distance and the shrinkage factor

	Talbot distance	Shrinkage factor
Theoretical	3.23 μm	—
SU-8 template	2.31 μm	29.2 ± 1.4%

It was found that the Talbot distance in the template shrank by 29.2% compared with the theoretical value. Similarly, previous studies have reported that the shrinkage of SU-8 nanostructures happened even in different feature sizes during the developing process [35, 42]. Thus, when fabricating precise and accurate 3D structures, it is important to consider the shrinkage factor.

**Fig. 2:**
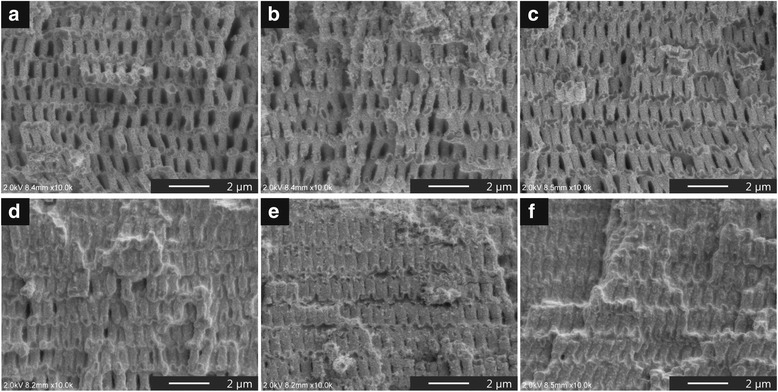
Cross-sectional SEM images of the ZnO precursor/polymer 3D structures. The infiltration process was conducted with different cycle numbers from one to six (**a**–**f**)

Figure 2 presents the cross-sectional SEM images of the ZnO precursor/polymer 3D structures fabricated by the infiltration process with different cycle numbers from one to six. As is apparent from the SEM images, the pre-baked ZnO precursor was uniformly distributed over the entire surface of the template in all cycle numbers, suggesting a conformal coating with no significant distortions or defects. There was a distinct difference between the results with and without precursor infiltration before the pre-baking process. A structural collapse was confirmed after pre-baking at 220 °C without pre-coated ZnO (Additional file 1: Figure S1c). It is evident that the pre-coated ZnO precursor plays a role as a protective layer to prevent a structural collapse caused by the shrinkage of the SU-8 template during the annealing despite one cycle of the infiltration process. Moreover, the pre-coated ZnO precursor became thicker by increasing the cycle number of the infiltration process and six cycles were enough to completely fill the 3D polymeric template with the pre-baked ZnO precursor. More importantly, we demonstrated ZnO precursor/polymer 3D structures with the same filling factor of the pre-coated precursor from the bottom to the top. In this paper, filling factor represents the proportion of the infiltrated precursor to the volume of the polymeric template after the infiltration process. So far, sol-gel and electrodeposition methods have been performed for uniform infiltrating of inorganic materials into a 3D template. However, those methods require high-density infiltration to obtain monolithic and freestanding inverse structures after template removal. Moreover, the materials for an inverse structure originate from the electrode side, which leads to a distribution gradient in the structure, especially in the latter method. Therefore, there have been few reports about a fabrication of nanoshell-based 3D inverse structures using a non-vacuum process with thickness controllability. In contrast, we achieved uniform infiltration with a relatively low filling factor by the proposed process and successfully demonstrated controllability for a thickness of the pre-coated precursor by the infiltration process.

**Fig. 3:**
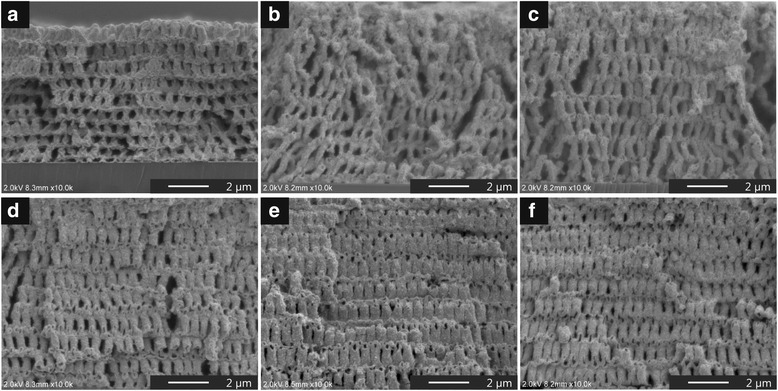
Cross-sectional SEM images of the resultant 3D inverse structures after post-baking**.** The infiltration process was conducted with different cycle numbers from one to six (**a**–**f**)

An additional baking process is needed to obtain a 3D ZnO inverse structure due to insufficient temperature since there is still a residual solvent in the pre-coated ZnO to remove the polymeric template in pre-baking. Thus, the post-baking at 410 °C for 4 h was performed with an electric furnace in oxygen atmosphere for template removal and pyrolysis for the pre-coated ZnO precursor to occur at the same time. Figure 3 indicates the cross-sectional SEM images of the resultant 3D inverse structures after post-baking. As a result, we obtained 3D inverse structures with pores in all cases where the cycle number of the infiltration process was set from one to six. However, the 3D inverse structures that precursor-infiltrated from one to three cycles showed some structural distortions, defects, and significant shortening in the out-of-plane direction. We consider that this shortening of the nanostructured film is caused by a deformation of the pre-coated precursor along the template with shrinkage resulting from the removal during the post-baking process. More importantly, the one-cycle-infiltrated structure possessed periodically gradient architectures with smaller structures in the bottom part toward the top side in an out-of-plane direction. This can be attributed to two reasons: (i) Template removal was initiated earlier in the bottom-side than the topside before solidification of ZnO; (ii) the bottom part of the inverse structure was compressively deformed under its own weight after template removal. These suggestions are consistent with the results in previous template process studies using SU-8 to obtain nanoshell-based 2D inverse patterns [43, 44], which have reported that the 2D inverse structural features can be changed depending on an intentional load and how the template was removed during baking process. In this study, we were also able to observe a deformation of the 3D inverse structure at connection areas with thinner thickness of the post-baked inverse layers. Moreover, it can be seen in the case of the structure of the conducted one-cycle infiltration (Additional file 1: Figure S2). This result suggests the possibility that the further detailed post-baking condition (i.e. temperature, rising and falling temperature profile) affects the structural features of a nanoshell-based 3D architecture with hierarchical structures.

**Fig. 4:**
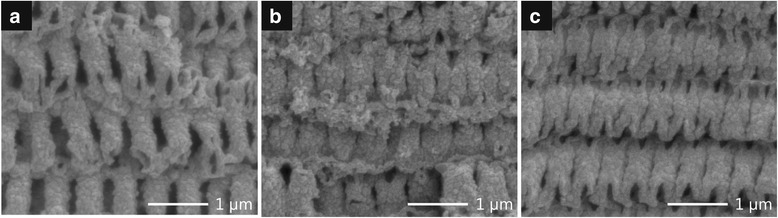
Cross-sectional SEM images with higher magnification of nanoshell-based 3D inverse structures. The infiltration process was conducted with different cycle numbers from four to six (**a**–**c**)

We demonstrated the fabrication of nanoshell-based 3D periodic structures with relatively low structural distortions and defects using four-cycle infiltration. Subsequently, the structure fabricated from a six-cycle infiltration possessed the most well-ordered structural periodicity. Figure 4 shows the cross-sectional SEM images with higher magnification of 3D inverse structures (Fig. 3d–f). Consequently, the nanoshell thicknesses within the 3D inverse structures with an infiltration from four to six cycles were <85, <100, and <125 nm, respectively. These results indicate that an incremental increase in the amount of infiltrated precursor by raising the cycle number of the infiltration process contributed to the preformation of the monolithic framework and a resultant 3D inverse structure with well-ordered periodicity derived from the template. Generally, high-density infiltration is a pre-requisite for a freestanding 3D structure to keep its periodicity in the non-vacuum process, thus forming a resultant structure with a specified filling factor depending on the template. In contrast, we successfully demonstrated the fabrication of nanoshell-based 3D periodic structures through the preformation of pre-baked precursor that works as a protective layer for the SU-8 template and a monolithic framework for an inverse structure as well. Interestingly, this process provides no excessive overlay on the template during the infiltration process, which inhibits the precursor solution from penetrating into the template. Since nanoshell-based 3D structures uniformly infiltrated, the ZnO precursor from the bottom to the top part was obtained via cycle-by-cycle sequential infiltration (Additional file 1: Figure S3). So far, thickness controllability of the nanoshell structure obtained by the proposed infiltration process is inferior to a method using ALD technique, which shows ability to provide atomic level accuracy and uniform surfaces. Although this limitation might narrow the application range, this problem could be improved by further progress of our process with optimal infiltration conditions. In addition, our process is a cost-effective, solution-based non-vacuum process which gives us a high impact compared to a vacuum process such as ALD, since vacuum process leads a costly-fabrication as well as a long processing time.

**Fig. 5:**
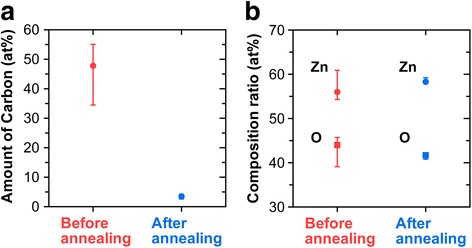
Differences of the amount of carbon and composition ratio of ZnO before and after post-baking. The **a** amount of carbon and **b** composition ratio of ZnO obtained by EDX analysis. These are the average values calculated from eight results detected in different observation points for both samples

We conducted EDX analysis to check whether the SU-8 template was removed and to identify a composition ratio of solution-derived ZnO after post-baking at 410 °C for 4 h. EDX analysis with an accelerating voltage of 5.0 kV was carried out on the cross-sectional structures for samples subjected to before and after post-baking (Additional file 1: Figure S4). In this measurement, we were able to identify peaks of ZnLα (1.025 keV), OKα (0.531 keV), and CKα (0.283 keV) derived from a SU-8 template and a solution-derived ZnO from the obtained EDX spectra. Figure 5 illustrates the differences in the amount of carbon and the composition ratio of zinc and oxygen, respectively. These are the average values calculated from eight results detected at different observation points for both samples. It is evident that the amount of carbon was significantly reduced from 47.8 to 3.5% through post-baking as shown in Fig. 5a, which means that the post-baking process was effective in template removal and the pyrolysis of the pre-coated ZnO precursor at the same time. The variation in the amount of carbon in before post-baking sample results from the fact that there was a difference in the exposed area of the template depending on the location. Figure 5b revealed that the composition ratio of the solution-derived ZnO after post-baking was 58.3:41.7 (Zn:O), which is almost the same as the value for ZnO nanorods fabricated by non-vacuum processes such as chemical bath deposition (CBD) [45] and hydrothermal method [46].

**Fig. 6:**
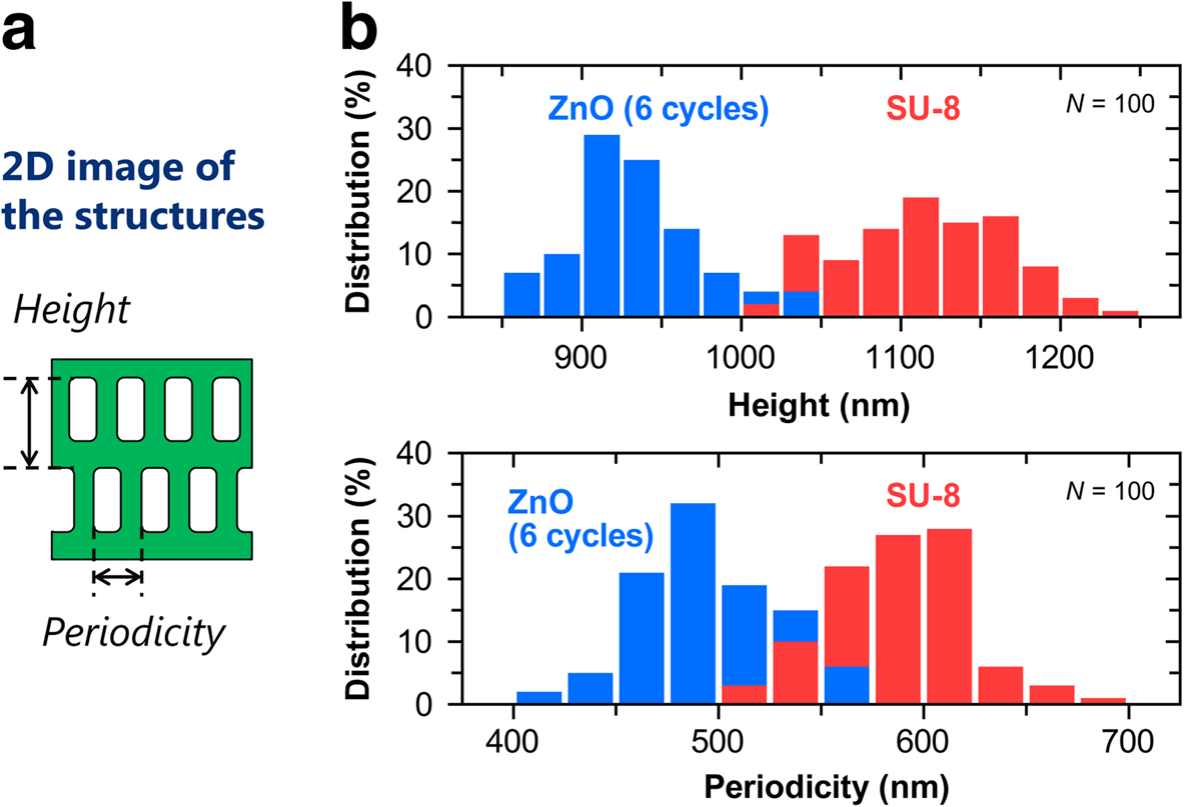
2D image structure and histograms that represent the measured values of the structural sizes. **a** Schematic diagram of 2D structure height and periodicity in in-plane direction of the structures and (**b**) histograms that represent the measured values of the structural sizes for the template and the inverse structure for ZnO and SU-8

In order to evaluate the shrinkage factor of the 3D ZnO inverse structure with the six-cycle infiltration in the templating process, the structure height and periodicity in the in-plane direction of the template and the inverse structure shown in Fig. 6a were measured from the cross-sectional SEM images. We created histograms representing the measured values of its structural sizes (Fig. 6b) and summarized average values and the calculated shrinkage factors in Table 2.

**Table 2:** Average values of structure height and periodicity in in-plane direction and the shrinkage factors

Structures	Height	Periodicity in in-plane direction
SU-8 template	1115 nm	589 nm
3D ZnO structure (6 cycles)	935 nm	495 nm
Shrinkage factor	16.1%	16.0%

From these results, the shrinkage factors of the structure height and in-plane direction periodicity were approximately 16% for both sizes. In this templating process, we believe that the shrinkage of solution-derived ZnO itself did not dominantly contribute to the shrinkage of the resultant 3D structure because the structural features strongly depend on the SU-8 template that works as a starting framework. Therefore, this indicates that the solution-derived ZnO shrinking has an effect on the thickness of ZnO nanoshells and does not affect the periodicity of the in-plane direction for the resultant structure. Thus, when fabricating precise and accurate 3D structures, it is important to consider the shrinkage factor. We compared the shrinkage factor of the structure height in this study to that in a similar study [23] using a polymeric template and TiO_2_ precursor. We discovered that our proposed process shows an improvement in the shrinkage factor of the structure height from 34% [23] to 16%. This improvement infers that pre-coated ZnO precursor plays an important role as a framework for inverse structure during post-baking.

The reflectance spectra of the polymeric template and the nanoshell-based 3D ZnO structure measured by UV-Vis spectroscopy (Additional file 1: Figure S5). The reflectance peaks of the template and the 3D ZnO structure were obtained at wavelengths of 410 and 450 nm, respectively. Although there is no reflectance peak that implies a creation of photonic bandgap, the reflectance peak similar to that of the template—which can be as high as 62%—was observed. We also evaluated the electronic bandgap of the ZnO fabricated by our proposed templating process from a measured transmission spectrum, in order to check whether ZnO was prepared from the aspect of an optical property. As a result, the electronic bandgap of ZnO that constitutes a nanoshell-based 3D structure was found to be 3.0 eV, which was determined from the (*αhν*)^2^ vs photon energy (*hν*) plot (Additional file 1: Figure S6). This value of the bandgap agrees well with that of ZnO nanorods fabricated by CBD method [47].

## Conclusions

We successfully performed a combination of proximity field nanopatterning and infiltration processes using solution-derived ZnO for nanoshell-based 3D periodic structure with structural flexibility and controllability. A novel infiltration process without defective colloidal templates resulted in 3D nanoshell structures, comparable to the structures formed from slow and expensive ALD process. Our study revealed the effect of the cycle number of infiltration process on the structural defects and sizes of resultant 3D ZnO structures. We demonstrated that a unique infiltration process is useful in the creation of a pre-formed layer that works as a protective layer for the template and framework for the inverse structure instead of ALD process. EDX analysis showed a drastic decrease in the amount of carbon in the structure after post-baking, indicating simultaneous template removal and pyrolysis of the pre-coated ZnO precursor. We also successfully achieved a significant improvement in the shrinkage factor of structure height compared to previous non-vacuum infiltration processes. Moreover, optical measurement for 3D ZnO structures clarified the bandgap of ZnO experimentally from the transmission spectrum. The nanoshell-based 3D periodic structure and our proposed process with high controllability and flexibility in the design of structural sizes have potential to be utilized for the further development of various applications including energy devices and sensors.

## Additional file

Additional file 1: Supplemental information. **Figure S1**: Cross-sectional SEM images of the structures. (a) 3D polymeric template as a starting structure, (b) sample post-baked at 400 °C for 1 h without pre-baking after precursor infiltration, and (c) the pre-baked template without precursor infiltration. **Figure S2**: Cross-sectional SEM images and schematic diagrams of the shrinkage models for one-cycle infiltrated structures. (a) After pre-baking, (b) the predicted model after post-baking, which indicates remaining pre-formed ZnO, and (c) after post-baking. **Figure S3**: Cross-sectional SEM images with lower magnification of 3D inverse structures. The infiltration process was conducted with different cycle numbers from one to six (a–f). **Figure S4**: A comparison of EDX analysis results. The differences in the results for (a) before and (b) after post-baking are apparent; inset illustrates cross-sectional SEM images of the structures and critical excitation potential for each element. **Figure S5**: Reflectance spectra of the polymeric template and the nanoshell-based 3D ZnO structure. **Figure S6**: (αhν)^2^ vs photon energy (hν) plot of nanoshell-based 3D ZnO structure. (DOCX 1903 kb)

## Abbreviations

3D: Three-dimensional
ALD: Atomic layer deposition
CBD: Chemical bath deposition
CVD: Chemical vapor deposition
DSSC: Dye-sensitized solar cells
EDX: Energy-dispersive X-ray spectrometry
MOD: Metal organic decomposition
PDMS: Polydimetylsiloxane
PhC: Photonic crystal
PnC: Phononic crystal
PnP: Proximity field nanopatterning
RIE: Reactive ion etching
SEM: Scanning electron microscopy
