# Supplementary material for: Fabrication of Nanoshell-Based 3D Periodic Structures by Templating Process using Solution-derived ZnO
Source: Nanoscale Res Lett. 2017 Jun 17;12:419. doi: 10.1186/s11671-017-2186-6 (PMC5474231; doi:10.1186/s11671-017-2186-6)
Supplement: Additional file 1: — Supplemental information. Figure S1: Cross-sectional SEM images of the structures. (a) 3D polymeric template as a starting structure, (b) sample post-baked at 400 °C for 1 h without pre-baking after precursor infiltration, and (c) the pre-baked template without precursor infiltration. Figure S2: Cross-sectional SEM images and schematic diagrams of the shrinkage models for one-cycle infiltrated structures. (a) After pre-baking, (b) the predicted model after post-baking, which indicates remaining pre-formed ZnO, and (c) after post-baking. Figure S3: Cross-sectional SEM images with lower magnification of 3D inverse structures. The infiltration process was conducted with different cycle numbers from one to six (a–f). Figure S4: A comparison of EDX analysis results. The differences in the results for (a) before and (b) after post-baking are apparent; inset illustrates cross-sectional SEM images of the structures and critical excitation potential for each element. Figure S5: Reflectance spectra of the polymeric template and the nanoshell-based 3D ZnO structure. Figure S6: (αhν)2 vs photon energy (hν) plot of nanoshell-based 3D ZnO structure. (DOCX 1903 kb) [file 11671_2017_2186_MOESM1_ESM.docx]

**Additional file 1**

**Title: Fabrication of Nanoshell-based 3D Periodic Structures by Templating Process using Solution-derived ZnO**

*Shinji Araki^1^, Yasuaki Ishikawa^*, 1^, Xudongfang Wang^1^, Mutsunori Uenuma^1^, Donghwi Cho^2^, Seokwoo Jeon^2^, and Yukiharu Uraoka^1^*

^1^Graduate School of Materials Science, Nara Institute of Science and Technology, 8916-5 Takayama, Ikoma, Nara 630-0192, Japan

^2^Department of Materials Science and Engineering, KAIST Institute for The Nanocentury, Korea Advanced Institute of Science and Technology, Daejeon 305-701, Republic of Korea

^*^E-mail: yishikawa@ms.naist.jp

**Figure S1 Cross-sectional SEM images of the structures.** (a) 3D polymeric template as a starting structure, (b) sample post-baked at 400°C for 1 h without pre-baking after precursor infiltration, and (c) the pre-baked template without precursor infiltration.


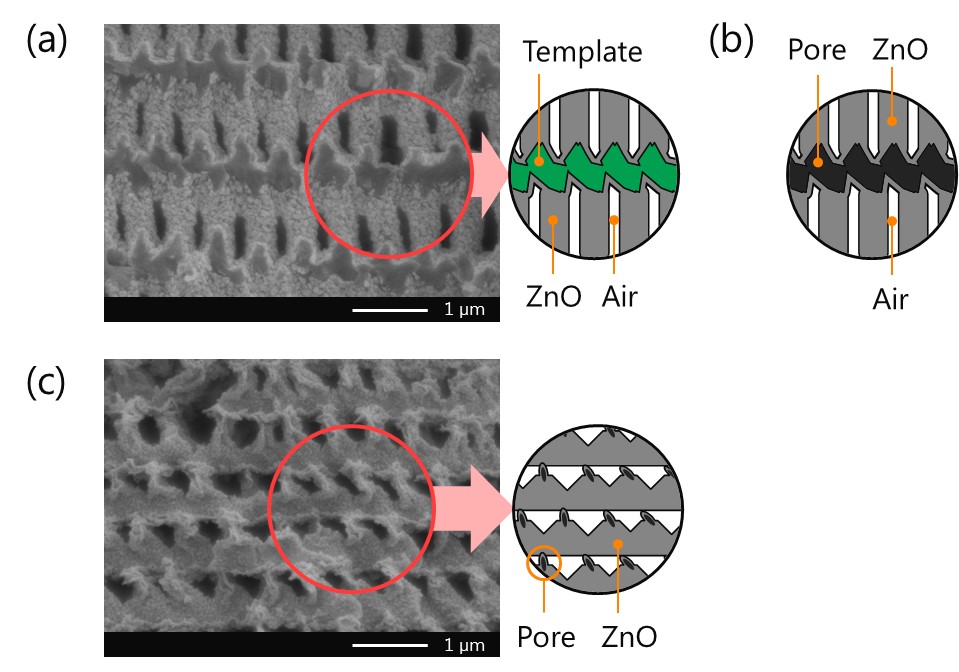


**Figure S2 Cross-sectional SEM images and schematic diagrams of the shrinkage models for one-cycle infiltrated structures.** (a) After pre-baking, (b) the predicted model after post-baking, that indicates remaining pre-formed ZnO, and (c) after post-baking.

**Figure S3 Cross-sectional SEM images with lower magnification of 3D inverse structures.** The infiltration process was conducted with different cycle numbers from one to six (a–f).

**Figure S4 A comparison of EDX analysis results.** The differences in the results for (a) before and (b) after post-baking are apparent; inset illustrates cross-sectional SEM images of the structures and critical excitation potential for each element.

Figure S5 Reflectance spectra of the polymeric template and the nanoshell-based 3D ZnO structure.


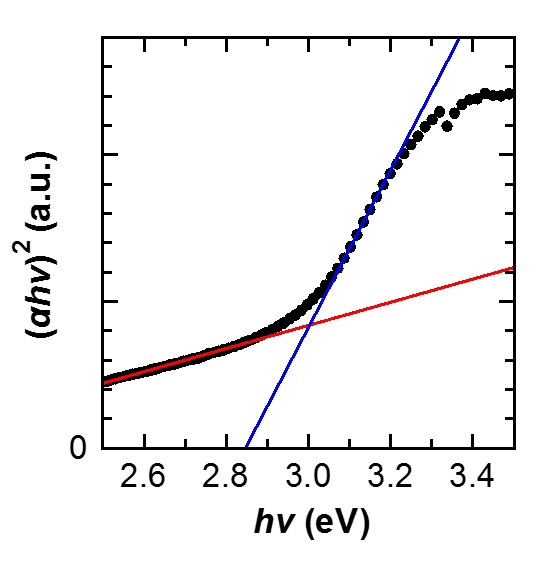


Figure S6 (*αhν*)^2^ vs photon energy (*hν*) plot of nanoshell-based 3D ZnO structure.
